# Engineering selection stringency on expression vector for the production of recombinant human alpha1-antitrypsin using Chinese Hamster ovary cells

**DOI:** 10.1186/s12896-015-0145-9

**Published:** 2015-06-02

**Authors:** Christine Lin Chin, Hing Kah Chin, Cara Sze Hui Chin, Ethan Tingfeng Lai, Say Kong Ng

**Affiliations:** Bioprocessing Technology Institute, Agency for Science, Technology and Research (A*STAR), Singapore, Singapore; Department of Pharmacy, Faculty of Science, National University of Singapore, Singapore, Singapore

**Keywords:** Expression vector, Selection stringency, Dihydrofolate reductase, Alpha1-antitrypsin, Internal ribosome entry site, Codon deoptimization

## Abstract

**Background:**

Expression vector engineering technology is one of the most convenient and timely method for cell line development to meet the rising demand of novel production cell line with high productivity. Destabilization of dihydrofolate reductase (dhfr) selection marker by addition of AU-rich elements and murine ornithine decarboxylase PEST region was previously shown to improve the specific productivities of recombinant human interferon gamma in CHO-DG44 cells. In this study, we evaluated novel combinations of engineered motifs for further selection marker attenuation to improve recombinant human alpha-1-antitrypsin (rhA1AT) production. Motifs tested include tandem PEST elements to promote protein degradation, internal ribosome entry site (IRES) mutations to impede translation initiation, and codon-deoptimized dhfr selection marker to reduce translation efficiency.

**Results:**

After a 2-step methotrexate (MTX) amplification to 50 nM that took less than 3 months, the expression vector with IRES point mutation and dhfr-PEST gave a maximum titer of 1.05 g/l with the top producer cell pool. Further MTX amplification to 300 nM MTX gave a maximum titer of 1.15 g/l. Relative transcript copy numbers and dhfr protein expression in the cell pools were also analysed to demonstrate that the transcription of rhA1AT and dhfr genes were correlated due to the IRES linkage, and that the strategies of further attenuating dhfr protein expression with the use of a mutated IRES and tandem PEST, but not codon deoptimization, were effective in reducing dhfr protein levels in suspension serum free culture.

**Conclusions:**

Novel combinations of engineered motifs for further selection marker attenuation were studied to result in the highest reported recombinant protein titer to our knowledge in shake flask batch culture of stable mammalian cell pools at 1.15 g/l, highlighting applicability of expression vector optimization in generating high producing stable cells essential for recombinant protein therapeutics production. Our results also suggest that codon usage of the selection marker should be considered for applications that may involve gene amplification and serum free suspension culture, since the overall codon usage and thus the general expression and regulation of host cell proteins may be affected in the surviving cells.

**Electronic supplementary material:**

The online version of this article (doi:10.1186/s12896-015-0145-9) contains supplementary material, which is available to authorized users.

## Background

The approval of tissue plasminogen activator produced in Chinese hamster ovary (CHO) cells in 1986 set the stage for CHO cells to become the dominant mammalian cell line for biopharmaceutical production till date. In addition to its ability to produce glycoproteins with post-translational modifications compatible to humans [1], and its refractory nature to human viruses [2], the availability of well-established gene amplification systems for CHO cells coupled with its ability to adapt and grow in serum-free suspension culture make CHO cells ideal for large scale high-titer cultures in the industry [3-6]. Currently, the titers of biopharmaceutical production from CHO cells have achieved gram per liter range and this 100 fold improvement since 1980s can be attributed to advances in bioprocess development, media development and cell line development. While many of these bioprocess and culture media improvements were kept as trade secrets [7], cell line development technologies like expression vector engineering, cell line engineering and clone screening technologies were extensively reviewed [1,7-10]. Typical cell line engineering strategy focuses on improving time integral of viable cell density (IVCD) and specific protein productivity (q_p_) of cells [11-16]. The availability of CHO genome data as well as the advancement of omics tools and *in silico* modelling of mammalian systems have also identified target genes with diverse array of functions to potentially improve the titer of biopharmaceuticals [9,17,18]. Together with the discovery of genome wide editing tools like zinc finger nucleases, transcription activator-like effector nucleases and meganucleases, more of these genes can be validated for their roles in biopharmaceuticals production [19-21].

To date, expression vector engineering technologies remain as the most timely and convenient method for new cell line development. The primary objective of expression vector engineering technologies is to improve the efficiency and efficacy of generating and isolating high producing clones. To increase the rate of transcription of gene of interest (GOI), the structure of chromatin can be altered by specific DNA elements that maintain the chromatin in an “open” state to increase transcription of the GOI. Examples of such elements are the ubiquitous chromatin opening element (UCOE) which is a methylation free CpG island [22], and the matrix attachment regions (MARs) which anchor the chromatin structure to the nuclear matrix during interphase [23]. As an alternative to altering the chromatin structure, site specific recombination is also used to introduce the GOI into a pre targeted genomic hotspot of the host cell line which was previously determined to enable stable and enhanced transcription of a reporter gene. Two site specific recombination systems, Cre and Flp, are well established and they are commonly used to insert GOI into targeted hot spots through their respective cis acting 34 bp loxP and 48 bp Flp Recombination Target (FRT) sites [24-30].

Another expression vector engineering approach is to improve selection stringency [31]. Selection stringency can be improved by using mutant neomycin phosphotransferase II selection markers with reduced affinities for the neomycin drug [32,33], by using a weak Herpes simplex virus thymidine kinase promoter [34], and by codon deoptimization of selection marker gene [35], which reduce the selection markers’ activity, transcription initiation and translation rate respectively. With a higher selection stringency, the selection marker gene has to be expressed at higher levels to be sufficient for surviving the selection process. As the GOI is likely integrated near the selection marker, this results in the high expression of the GOI to improve the probability of isolating high producing clones.

In addition to selection stringency, it is also important to co-localize the GOI with the selection marker, for efficient selection and successful amplification of the GOI gene [3,36]. While coexpression of GOI and selection marker using multiple promoters on the same vector may help in the co-localization, we have previously demonstrated that gene fragmentation can occurs at a high level of 14% during stable transfection of dual promoter dicistronic vector in CHO-DG44 cells [37]. As gene fragmentation dissociate the expression of selection marker with that of the GOI, additional cloning and screening steps are necessary for selection of high producing cell clones. To mitigate this, the GOI can be linked to the selection marker with the insertion of an internal ribosome entry site (IRES) [38]. By positioning the selection marker downstream of the GOI and IRES, the transcription of the selection marker is dependent on the successful transcription of the GOI upstream of it in the expression vector. Thus, the probability of selection marker expression without that of GOI and the survival rate of cells with fragmented transgenes are reduced.

In our previous studies, we have similarly shown that specific productivities can be improved when we increased selection stringency by destabilizing the selection marker through the addition of AU-rich elements (ARE) to promote mRNA degradation and murine ornithine decarboxylase (MODC) PEST region to enhance protein degradation [31]. Subsequently, an attenuated IRES element was used together with the PEST region to allow for high recombinant protein titer using stably amplified cell pools [39].

In this study, we evaluated various vector designs for further optimizing the strength of selection marker expression in CHO cells for the production of our model protein: recombinant human alpha1-antitrypsin (rhA1AT). Alpha1-antitrypsin is a serum protease inhibitor that protects tissues from enzymes secreted by inflammatory cells, and the protein is currently purified from human blood plasma as replacement therapy for patients who developed chronic obstructive pulmonary disease due to deficiency in the protein. rhA1AT expression vectors were constructed with tandem PEST sequence, further attenuation of the IRES element, and codon-deoptimization of the dihydrofolate reductase (dhfr) selection marker. The selection and amplification efficiency, recombinant protein productivity, relative transcript copy numbers and dhfr expression levels were then analyzed.

## Methods

### Expression vector construction

**Figure 1:**
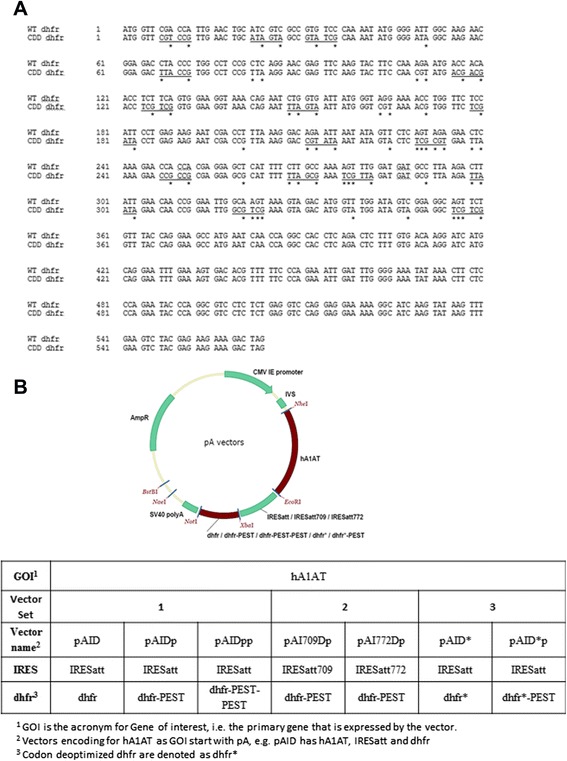
Illustrations of dhfr selection marker attenuation. **(A)** Nucleotide sequence of wild type (WT) and codon deoptimized (CDD) dhfr. Nucleotide changes are marked with * and new tandem codon pairs are underlined. **(B)** Vector map for vector sets with selection marker attenuation by destabilizing peptide, mutant IRES or codon deoptimization.

Human alpha-1-antitrypsin (hA1AT) precursor sequence (UniProt Identifier P01009-1, Accessed on 16 May 2012) was codon-optimized for CHO expression (Genscript, Piscataway, NJ). Attenuated Encephalomyocarditis virus (EMCV) IRES sequence (Clontech, Palo Alto, CA) was modified by deleting 7 nucleotides at position 709 to obtain IRESatt709, and by an A→C point mutation at position 772 to obtain IRESatt772 [40]. Murine dhfr sequence from pSV2-dhfr (ATCC, Manassas, VA) was modified by manual codon deoptimization to maximize the occurrence of de-optimized codon pairs in the first 120 amino acids of the sequence, based on *Cricetulus griseus* codon usage from database: http://www.kazusa.or.jp/codon/ [41] (Figure 1A). rhA1AT, IRESatt709, IRESatt772, codon-deoptimized dhfr (dhfr*), codon-deoptimized dhfr with PEST sequence (dhfr*-PEST), and murine dhfr with 2 tandem PEST sequences (dhfr-PEST-PEST) were synthesized by Genscript (Piscataway, NJ). These sequences were cloned into a mammalian expression vector backbone with a destabilized dhfr selection marker described previously [39,42], and a corresponding vector backbone with an unmodified dhfr selection marker to generate seven rhA1AT mammalian expression vectors, as illustrated in Figure 1B. Expression vectors were purified with NucleoBond® Xtra Midi EF (Macherey-Nagel) according to manufacturer’s instructions and dissolved in sterilized nucleases, proteases and pyrogen free biotechnology grade water (1st Base). The DNA quality and concentration was analysed using NanoDrop 2000 spectrophotometer (Thermo Scientific).

### Cell lines and cell cultivation

Suspension CHO-DG44 cells (Gibco™ Catalog number 12609–012, Invitrogen, Carlsbad, CA) adapted to HyQ PF-CHO (Hyclone, Logan, UT) with 4 mM L-glutamine (Invitrogen), 0.1% Pluronic® F-68 (Invitrogen) and 1× hypoxanthine and thymidine (HT) supplement (Invitrogen) were cultivated in 125 ml disposable Erlenmeyer flasks (Corning, Acton, MA) on shaker platforms set at 110 rpm in a humidified incubator at 37°C with 8% CO_2_. The cells were passaged every 3 to 4 days, and cell densities and viabilities were determined using an automated cell counter, Vi-Cell XR (Beckman Coulter, Fullerton, CA), according to manufacturer’s instructions.

### Transfection, selection and methotrexate (MTX) amplification

For transient transfections, suspension CHO-DG44 cells were seeded at a density of 3 × 10^5^ cells/ml into CD DG44 Medium (Life Technologies) with 8 mM L-glutamine (Invitrogen) and 0.18% Pluronic® F-68 (Invitrogen) in a 250 ml shake flask 3 days before transfection. For each transfection, 5 million cells were pelleted at 90 × g for 10 min at room temperature. The cell pellet was resuspended in the vector solution mix consisting of 2 μg of expression vector mixed with 100 μl of the supplemented Nucleofector™ Cell Line SG solution. This was transferred into a Nucleocuvette™ and electroporated with the Amaxa™ 4D-Nucleofector™ using pulse code DT-137 (Lonza). After 2 min, 500 μl of pre-warmed CD DG44 media was added, followed by incubation in a static incubator at 37°C with 5% CO_2_ (Sanyo) for 10 min, before the cells were transferred into 1.5 ml of prewarmed CD DG44 medium in a 24 well suspension culture plate. After 24 hours, cell pellets were harvested by centrifugation at 6000 × g for 10 min, washed once with sterile phosphate buffer saline (PBS) and stored immediately in a −80°C freezer for future analysis.

Generation of stable pools was performed on a Nucleofector I Device (Lonza), utilizing program U-24 and Nucleofector Kit V, as per manufacturer’s instructions. Briefly, each transfection was carried out using 1.5 × 10^6^ CHO DG44 cells and 4 μg of BstBI linearized rhA1AT expressing plasmid. The transfected cells were then resuspended with 2 ml HyQ PF-CHO containing 1 × HT supplement and 1% fetal bovine serum (FBS) (Life Technologies) and transferred into a 6-well adherent culture plate. At 48 h post-transfection, cells were detached, centrifuged at 100 × g rpm for 5 min and seeded at 2000 cells/well into a 96-well adherent culture plate containing the serum supplemented media without HT. Non-transfected cells died 7–14 days after selection. Selection efficiency was quantified as the percentage of wells that became confluent 4 weeks post-transfection. Gene amplification was subsequently induced in 30 randomly picked pools for each vector by passaging in serum media containing increasing concentrations of MTX (Sigma-Aldrich) from 10 to 50 nM MTX with each amplification step taking two week. The two highest producing cell pools from each vector at 50 nM MTX were selected for further MTX amplification to 300 nM MTX. The 50 and 300 nM MTX pools were then adapted to serum-free suspension culture in HyQ PF-CHO while maintaining the same MTX concentrations.

### Characterization of rhA1AT producing cell pools

Stably transfected cells pools that survived selection and MTX amplification at 10 and 50 nM MTX in 96 well plate adherent cultures were seeded into new 96 well plates for titer evaluation. These cells were allowed to grow for 14 days in a static incubator at 37°C with 5% CO_2_, with one change in culture medium at Day 7. Culture supernatants were harvested to determine rhA1AT titer.

Cells adapted to serum free culture were characterized by seeding them in 40 ml of serum-free medium at a cell density of 4 × 10^5^ cells/ml in 125 ml shake flask on shaker platforms set at 110 rpm in a humidified incubator at 37°C with 8% CO_2_. Cell densities and viabilities were determined daily using an automated cell counter, Vi-Cell XR (Beckman Coulter), according to manufacturer’s instructions, until culture viability dropped below 50%. Culture supernatant was sampled daily for analysis to determine rhA1AT titer, and for biochemical analysis using BioProfile 100 Plus (Nova Biomedical).

### Determining rhA1AT titer by enzyme-linked immunosorbent assay (ELISA)

The amount of rhA1AT in culture supernatants were determined using hA1AT specific ELISA quantification kit (Genway, #40-288-10066, USA) according to manufacturer’s instructions. Briefly, Nunc Maxisorp 96 well flat bottom plates (Thermo Fisher, USA) were coated with 2 μg/ml capture antibody in carbonate buffer at pH 9.6. For the hA1AT standard, 2-fold serial dilutions in sample diluent buffer (50 mM Tris, 0.14 M NaCl, 1% BSA, 0.05% Tween 20, pH 8.0) were prepared for a standard range of 1.4 to 90 ng/ml. rhA1AT samples were diluted in the range of 1:10000, 1:20000, 1:50000 and 1:75000 so that they fall within the range of the standard curve and were assayed in duplicates. 0.3 μg/ml horse radish peroxidase (HRP) conjugate secondary antibody in sample diluent buffer was used and 3,3′,5,5′-Tetramethylbenzidine (TMB), super slow for ELISA (T5569, Sigma, USA) served as the substrate. The absorbance at 450 nm was read using the ASYS UVM340 microplate reader (Biochrom, UK).

### Analysis of transcript levels by quantitative polymerase chain reaction (qPCR)

Total RNA was isolated from about 1 × 10^6^ cells using RNeasy® Mini kit (Qiagen) and treated with RNase-free DNase I (Qiagen cat. no. 79254) as per manufacturer’s instructions. Using the NanoDrop 2000 spectrophotometer (Thermo Scientific), RNA quantity and quality were determined by absorbance measurement at 260 nm and by the ratio of absorbance at 260 nm and 280 nm, respectively. 200 ng of total RNA was used for first-strand cDNA synthesis via ImProm-II™ Reverse Transcription System (Promega), with an oligo(dT)_28_ (Sigma-Aldrich) primer and treatment with recombinant RNasin® Ribonuclease Inhibitor, as per manufacturer’s instructions. RNA samples were stored in aliquots at −80°C and cDNA samples were stored at −20°C until use in qPCR.

qPCR were carried out on rhA1AT, dhfr and β-actin genes. Primer sets used for qPCR are described in Table 1. The qPCR reaction mixture was made up of 12.5 μl of SYBR® Green PCR Master Mix (Applied Biosystems), 2 μl of a primer mixture containing 5 μM of each forward and reverse primer, and 10.5 μl of cDNA template was loaded into each well of the MicroAmp® Optical 96-Well Reaction Plate (Applied Biosystems). Triplicates for each cDNA sample and buffer controls without template cDNA were loaded for each primer set. AB 7500 Fast Real-Time PCR System (Applied Biosystems) was used to analyse the samples using a thermal cycling condition that consisted of 50°C for 2 min, 95°C for 10 min followed by 40 cycles of 95°C for 15 sec and 60°C for 1 min. All run data was analysed using the Sequence Detection Software Version 1.4 (Applied Biosystems). A threshold fluorescence of 0.2 was used to determine the average threshold cycle (Ct) for all sample-primer sets. Results were normalized and analyzed using the comparative critical threshold (ΔΔCt) method [43]. Briefly, ΔCt for each sample was obtained by subtracting Ct of the target gene with Ct of the normaliser gene. ΔΔCt were then determined by subtracting the ΔCt of sample with ΔCt of the reference sample. The relative transcript copy numbers of the samples were calculated as 2^ΔΔCt^.

**Table 1:** **Sequences, amplicon sizes and sources of primer sets used for qPCR**

**Primer set**	**Primer sequences**	**Amplicon size (bp)**	**Sequence source**
rhA1AT	5′-GCAATGCTACCGCAATTTTCTT-3′	72	Primer Express Software 3.0
	5′-CATGGGTCAGCTCGTTTTCC-3′		(Applied Biosystems)
dhfr	5′-ACCAGGCCACCTCAGACT-3′	120	Ng et al., 2007 [31]
	5′-GAGAGGACGCCTGGGTATT-3′		
β-actin	5′-AGCTGAGAGGGAAATTGTGCG-3′	163	Fox et al., 2005 [52]
	5′-GCAACGGAACCGCTCATT-3′		

rhA1AT primer set was verified by sequencing the product obtained from PCR with the first-strand cDNA from a stably transfected CHO cell line as template.

### Analysis of proteins by Western blot

To probe for secreted rhA1AT in transiently transfected CHO-DG44 cells, culture media was harvested 3 days post-transfection and clarified by centrifuging at 18000 × g for 5 minutes, prior to Western blotting, as described below. For intracellular proteins (dhfr, β-actin and intracellular rhA1AT), 1 × 10^6^ DG44 suspension cells were lysed using 120 μl CelLytic M mammalian cell lysis/extraction reagent (Sigma, C2978, USA) supplemented with protease inhibitor cocktail without EDTA (Nacalai Tesque, #03969-21, Japan), as per manufacturers’ instructions. Solubilized proteins were collected using centrifugation at 18000 × g for 15 min. Total protein concentrations were determined using Pierce BCA protein assay reagent (Thermo Scientific, #23227, USA).

13 μl of clarified culture media or 20 μg total protein from each sample were resolved on 4-12% gradient PAGE gel (Thermo Fisher NuPAGE gel, # NP0321, USA). The proteins were transferred onto PVDF membrane using iBlot® (Thermo Fisher, #IB4010-01, USA) and then blocked for 1 h with a blocking buffer comprising of 1× Tris buffered saline with 0.1% Tween20 (TBST, First Base, Singapore) with 5% milk. The membrane was probed with primary antibodies against hA1AT (AAT (B9), 1:200 dilution, Santa Cruz, sc-59438, USA), dhfr (DHFR (49), 1:200 dilution, Santa Cruz, sc-136246, USA), or β-actin (ab8226, 1:2000 dilution, Abcam, United Kingdom) in blocking buffer overnight at 4°C. After washing with TBST, the membrane was incubated with 1:5000 diluted secondary antibody (Promega, W4021, USA) in blocking buffer for 1 h at room temperature. After washing, the bands were visualised using Amersham ECL Western blotting detection reagents and analysis system (RPN2106, GE Healthcare Life Sciences, USA) according to manufacturer’s instructions.

### Calculations

Cell doubling time (t_D_) was determined according to Equation 1, where μ is the specific growth rate.

1 $$ {t}_D=1\mathrm{n}\;2/\mu $$

Specific growth rate (μ) was determined as the slope of the linear trendline obtained by plotting ln(N) vs t according to Equation 2, where N is the viable cell density, N_0_ is the initial viable cell density and t is the culture time.

2 $$ \begin{array}{l}N={N}_0{e}^{\mu t}\\ {}1\mathrm{n}\left(\frac{N}{N_0}\right)=\mu t\end{array} $$

q_p_ was determined as the slope of the linear trendline obtained by plotting rhA1AT titer vs IVC according to Equation 3, where IVC is the cumulative integrated viable cell number, P is the cumulative rhA1AT produced and V is the culture volume.

3 $$ \begin{array}{l}{q}_p=\frac{1}{NV}\frac{dP}{dt}\\ {}{\displaystyle {\int}_0^pdP}={q}_p{\displaystyle {\int}_0^tNV\;dt}\\ {}P={q}_p\times IVC\end{array} $$

P was determined according to Equation 4 where p is the rhA1AT titer obtained from ELISA.

4 $$ {P}_t={P}_{t-1}+\left({p}_t-{p}_{t-1}\right)\times {V}_t $$

IVC was determined by trapezium rule according to Equation 5.

5 $$ IV{C}_t=IV{C}_{t-1}+0.5\left({N}_t+{N}_{t-1}\right)\times {V}_t\times \varDelta t $$

Student’s t-test was performed by comparing data from 2 vector sets by assuming the samples have unequal variance and using a one-tailed distribution, since we wanted to test whether the rhA1AT titers from cell pools transfected with the different vector sets were higher than that obtained using pAID vector.

## Results and Discussion

### Design of mammalian expression vectors with attenuated dhfr selection marker

In our previous study, we have demonstrated that selection marker attenuation with destabilizing sequences to reduce the transcript and/or protein levels of the selection marker gene, can improve the production of the recombinant gene of interest upon selection and MTX amplification [31]. An IRES element was then used for the production of recombinant sDectin-1, to allow for the successful MTX amplification of cell pools by transcriptionally linking the gene of interest and the selection marker [39]. With this design, selection stringency was maintained with the concurrent use of PEST destabilizing sequence for facilitating selection marker protein degradation and an attenuated IRES element for reduced translation of the selection marker.

In this study, we wanted to evaluate the effects of further selection marker attenuation on recombinant protein production in CHO cells using the IRES expression vector. As such, we designed 7 expression vectors expressing rhA1AT that can be classified into 3 sets (Figure 1B). Using rhA1AT as the gene of interest, the first vector set consists of pAID, pAIDp and pAIDpp. Comparing data from the use of pAIDp against pAID will allow us to validate the use of PEST element in improving stable recombinant gene expression, as observed in our previous studies [31,39]. The application of 2 tandem PEST elements in pAIDpp then allowed us to determine whether an additional PEST can further improve stable recombinant gene expression, as this has not been demonstrated in literature to our knowledge. The second vector set consists of pAI709Dp and pAI772Dp. These 2 vectors incorporated mutations described by Hoffman MA and Palmenberg AC [40] into the attenuated IRES [44,45]. This is to evaluate whether the further attenuation of selection marker expression with these additional impediment in translation initiation can improve stable recombinant gene expression. The third vector set comprised of pAID* and pAID*p. These 2 vectors incorporated a codon de-optimized dhfr selection marker (Figure 1A) to evaluate the use of codon deoptimization as a strategy to further reduce selection marker expression levels, since it will theoretically reduce translation efficiency, a different aspect of gene expression that is not affected by the attenuated IRES and PEST. Codon deoptimized dhfr has also been used by Westwood AD et al. [35] to improve recombinant protein yield in serum-containing CHO cell culture that were not exposed to MTX, though a different sequence is used in our study.

To verify the expression of rhA1AT, CHO-DG44 cells were transiently transfected with pAID and pAIDp vectors. For both transfections, rhA1AT protein was detected in the cell lysates and culture supernatants as respective weak and strong bands that were between the 50 and 75 kDa molecular weight markers (Additional file 1: Figure S1). This corresponded to the expected molecular weight of the glycoprotein at 55 kDa, while being bigger than the molecular weight of the full length polypeptide with and without the signal peptide at 46.7 and 44.3 kDa respectively. This suggests that the rhA1AT from CHO cells is secreted and glycosylated, as previously reported by Lee KJ et al. [46]. While we also attempted to probe for intracellular dhfr protein from transiently transfected cells, it was below the detection limit by Western blotting for all our vectors. Nonetheless, rhA1AT and dhfr transcript levels of transfected cells were more than 8 fold higher than that of the untransfected control by qPCR, indicating that all the vectors can be transcribed successfully.

### Selection and MTX amplification efficiencies for rhA1AT production

Stable transfections of the rhA1AT expression vectors with modified selection markers were subsequently performed by plating transfected CHO-DG44 cells in 96 well plates for selection in a HT-deficient (−HT) culture medium. 30 randomly picked cell pools for each vector from these 96 well plates were then subjected to sequential MTX amplification at 10 nM and 50 nM concentrations. The selection and amplification efficiencies are listed in Table 2. With selection in –HT medium, pAID, pAIDp, pAI772Dp and pAID* gave high selection efficiencies of more than 80%, pAIDpp gave 70%, pAID*p gave 45%, and none of the pAI709Dp or untransfected control cell pools survived. This suggests that the further attenuation of the IRES element in pAI709Dp did not allow for ample dhfr expression for cells to survive the selection in –HT medium. In addition, tandem PEST and codon deoptimization, in pAIDpp and pAID*p vectors respectively, have the effects of further lowering selection efficiency, compared to pAIDp vector, which suggest that dhfr expression is lowered in these vectors. At 10 nM MTX, the reduced survival fitness of the pAIDpp and pAID*p vector population is also shown by the markedly lower amplification efficiency of these samples as compared to the other vector sets. The trend in survival fitness was more obvious when the cells were subjected to a greater selection pressure of 50 nM MTX, where amplification efficiencies were of the order pAID > pAIDp > pAID* > (pAI772Dp and pAIDpp) > pAID*p > pAI709Dp. This shows that additional selection marker attenuation acted to reduce selection fitness of the transfected cells, for example, pAID > pAIDp; pAIDp > pAI772Dp, pAIDpp, pAID*p and pAI709Dp; and pAID* > pAID*p.

**Table 2:** **Selection and amplification efficiency of different vectors**

**Vector**	**-HT Selection efficiency (%)** ^**1**^	**10 nM MTX Amplification efficiency (%)** ^**2**^	**50 nM MTX Amplification efficiency (%)** ^**2**^
pAID	100	87	73
pAIDp	97	77	40
pAIDpp	70	40	10
pAI709Dp	0	0	0
pAI772Dp	86	90	13
pAID*	84	100	23
pAID*p	45	7	3
Untransfected control	0	0	0

^1^Total number of cell pools subjected to –HT selection is 96.

^2^30 cell pools that survived –HT selection were randomly selected for sequential MTX amplification at 10 nM followed by 50 nM. Amplification efficiency is calculated as the percentage of cell pools out of the 30 initial cell pools that survived the amplification process.

**Figure 2:**
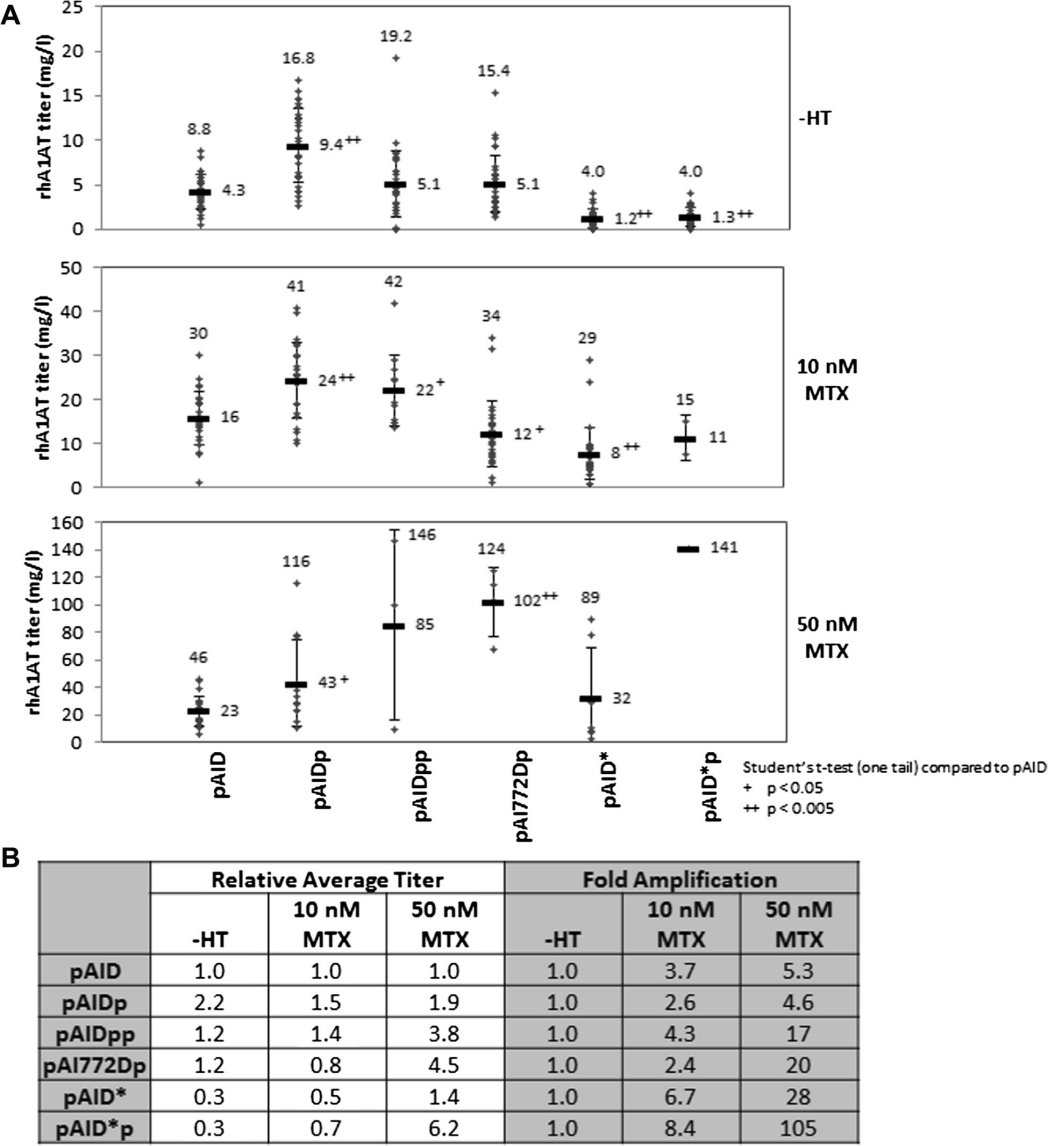
Recombinant human A1AT titers of mini pools derived from different expression vectors in 96 well-plate cultures. Cells are seeded into 96 well plate and allowed to grow for 14 days with one change in culture medium at Day 7. Culture supernatant was then harvested for hA1AT ELISA to determine recombinant protein titer. **(A)** rhA1AT titers of each cell pool at different stages of selection and MTX amplification were plotted. Titers of the highest producing cell pool and the average titer of all analyzed cell pools were annotated on the graphs. The average titers of each vector set were compared to that of pAID at the same amplification stage and analyzed by one-tail Student’s t-test. Results of the analysis were annotated besides the average titers as + for p < 0.05 and ++ for p < 0.005. **(B)** Relative average titers and Fold amplification of the cell pools. Relative average titers of each set of cell pools were calculated by dividing the average titers with that of the pAID set. Fold amplification were calculated by dividing the average titers of each set of cell pools with that of the same cell pool set after –HT selection.

The 30 randomly selected cell pools for each vector that were subjected to sequential MTX amplification were then evaluated for rhA1AT production. The cell pools were re-plated into a new 96 well plate and cultured for 14 days. After which, the culture supernatant was harvested to determine the rhA1AT titers. The titers obtained using the different expression vectors are illustrated in Figure 2. While there is some variability in rhA1AT titers regardless of vector used, the average titers of the pAIDp vector are 1.5 to 2.2 fold higher than those of the pAID vector at various amplification stage (Figure 2, p value < 0.05). This concurs with our previous report [31] and provided further support to the hypothesis that selection marker attenuation can improve recombinant protein production in the MTX amplification system.

With additional selection marker attenuation by tandem PEST (pAIDpp) and further IRES attenuation (pAI772Dp), it was surprising that the titers in –HT medium were similar to that of pAID vector. Improvements in titers for pAIDpp and pAI772Dp were only observed in the amplification media with 10 nM MTX and 50 nM MTX respectively. These improvements in rhA1AT titers corresponded with the decrease in the amplification efficiencies of the respective cell pools (Table 2). We thus speculate that the additional dhfr attenuation may have negatively impacted recombinant protein production at the onset of selection. rhA1AT titers then increased when the cells are exposed to MTX, driven by the increased selection stringency and potential increases in gene copy number induced by MTX. With fold amplifications of 17 and 20 at 50 nM MTX for the pAIDpp and pAI772Dp vectors respectively, the effect of selection stringency became dominant for these cell pools to obtain average rhA1AT titers 3.8 to 4.5 fold higher than that of pAID, higher than the improvement driven by the pAIDp vector (Figure 2B).

As for the vectors with partial dhfr codon deoptimization (pAID* and pAID*p), these gave rhA1AT titers lower or similar to that from pAID cell pools in both the –HT and 10 nM MTX media. These low rhA1AT titers are obtained despite the higher selection stringency as demonstrated by the relatively higher fold amplifications (Figure 2B) and the lower selection and amplification efficiencies of the pAID*p vector (Table 2), in contrast to the study by Westwood AD et al. [35] where higher selection stringency led to higher recombinant protein titers in serum-containing –HT medium. To understand how the different codon de-optimized dhfr sequences used may have contributed to the different observations, we compared our codon de-optimized dhfr with the 2 codon de-optimized dhfr used by Westwood AD et al. [35] and the wild-type murine dhfr: the codon adaptation indexes (CAI) of these dhfr were calculated with an online CAI calculator [47] to be 0.586, 0.529, 0.437 and 0.741 respectively, based on Cricetulus griseus codon usage from database: http://www.kazusa.or.jp/codon/ [41]. The CAI for the first 120 amino acids of our dhfr which were codon de-optimized was 0.500, lower than that of the full protein, although it is still higher than one of the sequences used by Westwood AD et al. [35]. This suggests that the codon deoptimized dhfr used in both studies may be comparable. However, in addition to the codon deoptimized dhfr, we are also applying an attenuated IRES to further weaken the expression of dhfr in our study. Hence, we speculate that this additional dhfr attenuation in the pAID* and pAID*p vectors may have further reduced dhfr expression to negatively impact recombinant protein production, similar to pAIDpp and pAI772Dp cell pools.

At 50 nM MTX, the effect of higher fold amplification became dominant when the cell pools from pAID* and pAID*p vector sets separate into 2 clusters: one low producing cluster comprising of 5 pAID* pools producing low titers of rhA1AT (2.8 – 29 mg/l), and the other high producing cluster comprising of 2 pools from pAID* and 1 from pAID*p that are producing high titers of rhA1AT (78 – 141 mg/l). As the 3 cell pools from the high producing cluster were derived from the top 3 producer cell pools in these vector sets at 10 nM MTX, we postulate that these cell pools may have transgene integration into sites where gene amplification may be facilitated. As such, gene amplification triggered by the higher MTX concentration may be used by these cells as the primary means of rescue from the stringent selection to give rise to the higher rhA1AT titers in these cultures.

### Growth and productivity evaluation of top cell pools

The top 2 producing cell pools from each vector set at 50 nM MTX were subjected to further amplification to 300 nM MTX. Both sets of 50 nM MTX and 300 nM MTX cell pools were adapted to serum free suspension culture to obtain their growth and rhA1AT production profiles in batch shake flask cultures (Table 3, and Additional file 2: Figure S2). As only 1 cell pool from vector pAID*p survived the amplification process, and 1 cell pool for vector pAID survived the adaptation to suspension culture, these vector sets were only represented by 1 cell pool in this evaluation.

**Table 3:** **Growth and rhA1AT productivities of top 2 cell pools from each vector set** ^#^

**MTX concentration**	**Vector**	**Pool 1**				**Pool 2**				**Average**		
		**Max titer** ^**%**^ **(mg/l)**	**q**_**p**_ **(pcd)**	**t**_**D**_ **(h)**	**Fold titer increase** ^**+**^	**Max titer** ^**%**^ **(mg/l)**	**q**_**p**_ **(pcd)**	**t**_**D**_ **(h)**	**Fold titer increase** ^**+**^	**Max titer** ^**%**^ **(mg/l)**	**q**_**p**_ **(pcd)**	**t**_**D**_ **(h)**
**50 nM**	pAID	256	17.6	46	5.6					256	17.6	46
	pAIDp	492	25.7	36	4.2	244	10.9	36	3.1	368	18.3	36
	pAIDpp	647	29.0	33	4.4	539	25.9	44	5.4	593	27.5	39
	pAI772Dp	1054	41.3	28	8.5	937	33.8	26	8.2	996	37.6	27
	pAID*	275	14.4	41	3.1	170	7.8	40	2.2	222	11.1	40
	pAID*p	277	8.4	33	2.0					277	8.4	33
**300 nM**	pAID	560	32.5	34	2.2					560	32.5	34
	pAIDp	514	25.8	33	1.0	240	13.1	37	1.0	377	19.5	35
	pAIDpp	1146	88.2	45	1.8	846	41.9	41	1.6	996	65.0	43
	pAI772Dp	1111	48.6	42	1.1	863	35.4	27	0.9	987	42.0	34
	pAID*	721	34.3	43	2.6	398	22.1	33	2.3	559	28.2	38
	pAID*p	412	17.7	29	1.5					412	17.7	29

^#^The 2 cell pools that gave the highest titers in 96 well-plate adherent culture with 50 nM MTX were chosen for further amplification to 300 nM MTX. The 50 nM and 300 nM MTX cell pools were then adapted to serum-free suspension culture in shake flasks to evaluate their growth and rhA1AT production profiles. The top producing cell pool in 96-well plate format (Figure 2) is indicated as Pool 1. Only 1 cell pool from vectors pAID and pAID*p survived the adaptation to suspension culture or the MTX amplification process respectively.

^%^Maximum titer was taken as the highest rhA1AT titer assayed from the first 11 days of the batch culture.

^+^Fold titer increase for cell pools in 50 nM MTX suspension culture was compared against the titers of the same cell pools in adherent 96 well plate cultures, whereas that for cell pools in 300 nM MTX suspension culture was compared against the titers of the same cell pools in 50 nM MTX suspension culture.

Comparing the 50 nM MTX cell pools in suspension shake flask culture (Table 3) and adherent 96-well plate cultures (Figure 2), the rhA1AT titers from the suspension cultures were 2.0 to 8.5 fold higher than those from the 96-well plate cultures. This improvement is likely attributable to the higher cell densities of the suspension cultures. It is nonetheless interesting to note that the cell pools derived using the same vector were somewhat consistent in their fold increase in maximum rhA1AT titers, suggesting that these titer improvements due to adaptation to serum free suspension cultures may be characteristics of the different expression vectors. In addition, we noted that both pAI772Dp cell pools have the highest improvement in maximum titers at 8.5 and 8.2 folds, much higher than the 2.0 to 5.6 fold improvements from cell pools derived using other vectors. This suggests that the cell pools derived using the pAI772Dp vector performed much better in serum free suspension culture. The exponential doubling times of these cell pools are also the lowest at 28 and 26 h respectively. These factors resulted in maximum rhA1AT titers of 1.05 and 0.94 g/l for these cell pools in shake flask batch culture. This is noteworthy because we have achieved g/l titers of a recombinant protein using a 2-step MTX amplification that took less than 3 months, and that this titer was obtained from shake flask batch culture of mammalian cell pools in un-optimized culture medium.

While some cell pools adapted to serum free suspension culture successfully, others did not fare too well: the maximum titers for pAIDp Pool 2, both pAID* cell pools and pAID*p cell pool increased by only 2.0 to 3.1 folds, much lesser than that of pAID at 5.6 fold. These cell pools also have lower exponential specific rhA1AT productivity rates compared to pAID, though they have shorter exponential doubling times. As such, despite the higher titers observed in adherent 96-well plate cultures, the maximum titers from these cell pools became comparable to that of the pAID cell pool after adaptation to serum free suspension culture at 50 nM MTX, with pAID* Pool 2 giving a maximum titer lower than pAID.

Of the 4 cell pools that did not adapt well into suspension serum free culture, 3 of which contained the codon-deoptimized dhfr selection marker. This suggests that the selection marker codon-deoptimization may be detrimental to recombinant protein productivity in suspension serum free culture of MTX amplified cells. As codon usage changes to improve survivability of single cell organisms and viruses have been previously reported and discussed [48-51], we postulate that this observation may be due to codon usage changes in these cells to improve survivability under serum free selective conditions. Clonal differences, such as changes in the cell epigenetics during adaptation and overall cell fitness in serum free suspension culture, may also have contributed to these observations.

Comparing between 50 nM MTX and 300 nM MTX suspension cell pools, cell pools derived using the same vector were also consistent in their fold increase in maximum rhA1AT titers, suggesting that the titer improvements by MTX amplification may also be characteristics of the different expression vectors. For the cell pools adapted to 300 nM MTX, we observed that maximum titers for pAIDp and pAI772Dp cell pools maintained at the similar levels exhibited by their corresponding 50 nM MTX suspension culture, suggesting that no amplification occurred for these cell pools in this step of MTX amplification. Similar observations had been made from our previous experience [39] during MTX amplification of our production cell pool. We postulate that for each step of MTX increase, a critical MTX concentration needs to be reached prior to the occurrence of gene amplification in the cells. Seemingly, the postulated critical MTX concentration for these 4 cell pools transfected with the pAIDp or pAI772Dp vectors was not crossed at 300 nM MTX, resulting in similar cell characteristics compared to the corresponding 50 nM MTX cell pools. In contrast, the pAID*p and pAIDpp cell pools gave 1.5 to 1.8 fold increases in maximum titers, followed by the pAID and pAID* cell pools with fold increases of 2.2 to 2.6. These fold increases in maximum titers were due to corresponding improvements in specific rhA1AT productivities, as indicated in Table 3. This observation also suggests that the MTX amplification step up from 50 nM to 300 nM MTX may be more suitable for pAID*, pAID, pAIDpp, and pAID*p vectors, in that order, and not so suitable for pAIDp and pAI772Dp vectors. As a result of more amplification, the pAIDpp Pool 1 also attained a g/l maximum titer of 1.15 g/l, comparable to that of the pA772Dp Pool 1 at 1.11 g/l. To our knowledge, this is the highest g/l recombinant protein titers reported from shake flask batch culture of stable mammalian cell pools.

This study thus illustrates the importance of expression vector optimization for the generation of high producing stable cell lines that are essential for the manufacturing of recombinant protein therapeutics. In addition, a relatively quick development of high producing stable cell pools, such as the system demonstrated in this study, can also be a viable alternative for the rapid production of milligrams to grams of representative product for preclinical development of therapeutic proteins, a niche that is commonly fulfilled with transient transfection of HEK293 cells: Besides the ease of scale up and having an identical expression system as the final product, the ability to generate stable cell pools with high productivities as described here will also mean that less scale up is required to obtain the desired gram quantities of representative product.

### Transcript and protein level characterization of cell pools

**Figure 3:**
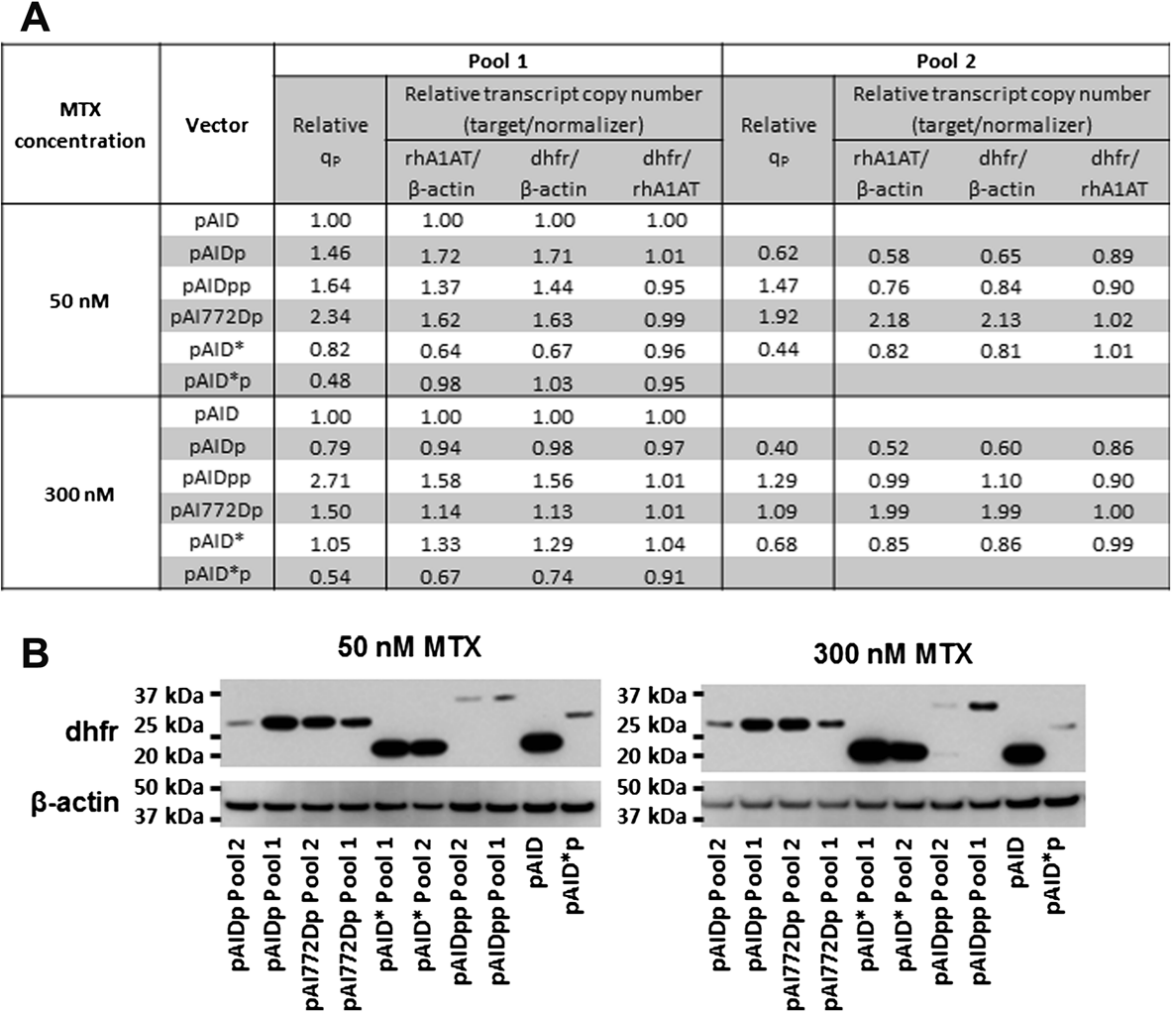
Relative transcript copy numbers and Western blot of amplified suspension cell pools. Exponentially growing cells were harvested from shake flask cultures of the 50 nM and 300 nM MTX amplified suspension cell pools from each vector set. **(A)** First-strand cDNA from each sample were analyzed by qPCR. Threshold cycle (Ct) data were analyzed using the ΔΔCt method using pAID as sample reference and β-actin as normalizer. Relative transcript copy numbers were calculated as 2^ΔΔCt^. Standard deviations from technical triplicates were determined to be lesser than 10% of the relative values. The relative specific productivity q_p_ was obtained by normalizing exponential q_p_ to that of pAID at the respective MTX concentrations. **(B)** 20 μg of total protein from each sample were resolved by SDS-PAGE, transferred onto a PVDF membrane, and probed with primary antibodies against dhfr and β-actin.

To gain some insights on how the different vectors resulted in the different phenotypes, we analysed the relative transcript copy numbers, as well as the relative dhfr protein expression, in the 50 nM and 300 nM MTX cell pools from suspension shake flask cultures (Figure 3).

To derive the relative transcript copy numbers, qPCR results of rhA1AT and dhfr were normalized with the housekeeping gene β-actin to determine their relative expression levels compared to the pAID cell pool (Figure 3A). We first observed that the relative transcript copy numbers of rhA1AT and dhfr were similar in each cell pool. This was further demonstrated when we derived the relative transcript copy numbers of dhfr with rhA1AT as the normalizer, to obtain similar numbers ranging from 0.86 to 1.04 (Figure 3A). This transcriptional correlation of the rhA1AT and dhfr genes verified the successful use of a single promoter and IRES mediated translation to physically link these genes on the same transcript with the use of these different vectors, even though the cells were subjected to MTX amplification.

Next, we observed that cell pools with relative q_p_ higher than that of the pAID cell pool also have higher rhA1AT transcript copy number, with the exception of pAIDpp Pool 2 which suggests that its translation efficiency of the rhA1AT gene is higher (Figure 3A). However, there is no general correlation between relative q_p_ to rhA1AT transcript copy number: for example when the pAI772Dp cell pools at 50 nM MTX were compared, Pool 1 has a lower rhA1AT transcript copy number but a higher relative q_p_ compared to Pool 2. This suggests that the overall productivity of rhA1AT was not solely dependent on transcript levels, but possibly other factors such as translation and secretion efficiencies, which may be peculiar to individual cell pools and not vector specific. Nonetheless, with the pAI772Dp and pAIDpp vectors clearly showing superior q_p_ and maximum titers, we postulate that each cell pool may have underwent varying extent of changes in these different factors during the MTX amplification process, to attain the levels of productivities dictated by the use of the different expression vectors.

In the same comparison of relative q_p_ to relative transcript copy number, we noted that both parameters decreased for the pAIDp and pAI772Dp cell pools when these cell pools were amplified from 50 nM MTX to 300 nM MTX. In view of the 1.8 and 2.2 fold respective increases in q_p_ and maximum titers of the pAID cell pool used for normalization, this verified our previous postulate that the rhA1AT transgene had minimal gene amplification and as such lowered relative transcript copy numbers, in the 4 pAIDp and pAI772Dp cell pools during the increase in MTX concentrations from 50 nM to 300 nM MTX.

Similar to the rhA1AT, we observed that the relative dhfr transcript copy numbers varied between the cell pools, even between those generated using the same expression vector (Figure 3A), although all these cell pools were able to survive at their respective MTX concentrations. This suggests that the overall activity of dhfr to allow for the survival of these cell pools was also not completely dependent on transcript levels, but other factors such as translation efficiency, dhfr protein stability and activity. Examining the cell pools with relative dhfr transcript copy numbers greater than 1.1 as compared to pAID, there are 4 such cell pools at 50 nM MTX (pAIDp Pool 1, pAIDpp Pool 1, pAI772Dp Pools 1 and 2) and 300 nM MTX (pAIDpp Pool 1, pAI772Dp Pools 1 and 2, and pAID* Pool 1). When the intracellular dhfr proteins were analysed, we noted that these higher relative dhfr transcript copy numbers did not translate to higher dhfr protein levels when compared to pAID with the only exception of pAID* Pool 1 at 300 nM MTX, and that the pAID dhfr bands were one of the darkest at both 50 nM and 300 nM MTX (Figure 3B). This demonstrates that the strategies of further attenuating dhfr translation efficiencies and protein stabilities, with the use of the mutated IRES and tandem PEST respectively, were effective in reducing dhfr protein levels in these MTX amplified cell pools: Specifically comparing the dhfr protein levels in pAID, pAIDp and pAIDpp cell pools, they were generally in decreasing intensity despite the variability in their relative dhfr transcript copy numbers, suggesting that the tandem application of PEST is effective in decreasing the dhfr protein stability. Comparing the pAIDp and pAI772Dp cell pools, although pAID772Dp Pool 2 had relative dhfr transcript copy numbers much higher than that of pAIDp Pool 1, their dhfr protein level seem comparable. On the other hand, pAI772Dp Pool 1 which had relative dhfr transcript copy numbers similar to that of pAIDp Pool 1 had comparable dhfr protein levels at both 50 nM and 300 nM MTX. (Figure 3) These evidences suggest that the IRESatt772 mutant was effective in reducing translation efficiency.

On the contrary, the dhfr protein levels of pAID* suggest that the codon deoptimization applied in this study was not effective in reducing dhfr protein expression in suspension serum free culture, since the pAID* dhfr protein bands were of similar intensities to the corresponding pAID band, despite the lower transcript levels for both pAID* cell pools at 50 nM MTX and for pAID* Pool 2 at 300 nM MTX. With a higher relative dhfr transcript copy number, pAID* Pool 1 had a dhfr protein band that was also darker than that of pAID. These observations suggest that host cell codon usage had changed in these cell pools. This concurs with our previous postulate that codon usage changes in these cell pools may have enhanced their survivability in suspension serum free culture under selective conditions, and that these changes might have negatively affected rhA1AT productivity during adaptation to suspension serum free culture, since the rhA1AT gene was codon-optimized for CHO expression. We noted that previous observations in serum containing media suggest that codon deoptimization is effective in improving selection stringency and the titer of the recombinant protein product: These include our previous observations of lower amplification efficiency (Table 2) and higher titers of pAID* cell pools in 96 well plate serum containing culture (Figure 2), and the lower dhfr levels measured with the use of codon deoptimized dhfr in the previous study by Westwood AD et al. [35]. Nonetheless, we noted that these are not inconsistent with our observed ineffectiveness of this strategy in suspension serum free culture: we postulate that host cell gene expression and regulation may have become less important to cell survivability during the adaptation to serum free suspension culture, thus allowing changes to host cell codon usage to improve overall cell fitness with better expression of the selection marker in the serum free selective environment, since serum supplemented CHO cell culture is known to grow faster and has different gene expression profile compared to serum-free CHO culture. In an attempt to extend this concept of a changing host cell codon usage to cell biology studies, we noted that this may not be applicable to the common usage of microbe-derived selection markers since most of such cultures are in the presence of serum. Another consideration that may be peculiar to our study is that in our cell pools, the translation of the selection marker may become limiting to cell survival due to the MTX-driven gene amplification. This may be an additional driving force for codon usage changes in these cells to improve cell fitness and survivability. Nonetheless, this result suggests that codon usage has to be carefully considered for applications in genes that may affect survivability of cells, since the overall codon usage in the surviving cells, and thus the general expression and regulation of host cell proteins may be affected.

Lastly, we noted that the molecular weights of the dhfr proteins (Figure 3B) corresponded with their expected molecular weights of 21, 26 and 30 kDa for the dhfr without PEST, with a single PEST and with tandem PEST sequences respectively. While we have also sequenced the transcript from these cell pools to verify that these cells were indeed transfected with the said expression vectors (data not shown), this protein level data demonstrates that these cell pools were also producing the respective dhfr proteins with the engineered modifications.

## Conclusions

We evaluated the effect of further selection marker attenuation using novel combinations of attenuation strategies to improve recombinant protein production using rhA1AT as our model protein. 7 expression vectors expressing rhA1AT were constructed to introduce the application of 2 tandem PEST elements in pAIDpp, further attenuation of the IRES element in pAI709Dp and pAI772Dp, and incorporation of a codon de-optimized dhfr selection marker in pAID* and pAID*p. pAIDpp and pAI772Dp vectors gave further improvements in rhA1AT production when compared to pAID and pAIDp vectors, indicating that further selection marker attenuation can improve recombinant protein production. Using the pAI772Dp vector, we generated a cell pool that gave a maximum titer of 1.05 g/l of rhA1AT in an un-optimized shake flask batch culture using a 2-step amplification till 50 nM MTX that took less than 3 months. Using the pAI772Dp and pAIDpp vectors, we generated cell pools that gave a maximum titer of 1.11 and 1.15 g/l respectively in un-optimized shake flask batch cultures at 300 nM MTX. To our knowledge, this is the highest reported recombinant protein titer from shake flask cultures of stable mammalian cell pools. This system thus provide a means for the rapid development of high producing stable cells that can be used for the manufacturing of recombinant protein therapeutics, or for the rapid production of representative product for preclinical development of therapeutic proteins.

To gain some insight on the mechanism of this phenomenon, we measured the relative transcript copy numbers of rhA1AT and dhfr genes, and analyzed the dhfr protein expression in the cell pools. The relative transcript copy numbers demonstrated that the transcription of rhA1AT and dhfr genes were correlated due to the IRES linkage, although the results also suggested that the protein expression were not solely dependent on transcript levels. Protein level analysis of dhfr validated that the cell pools were indeed expressing the modified dhfr of the correct molecular weight. In addition, it showed that the strategies of further attenuating dhfr protein expression with the use of a mutated IRES and tandem PEST, but not codon deoptimization, were effective in reducing dhfr protein levels in these MTX amplified cell pools in suspension serum free culture. Our data also suggests that codon usage of the selection marker should be considered for applications that involve gene amplification and serum free suspension culture, since the overall codon usage and thus the general expression and regulation of host cell proteins may be affected in the surviving cells.

## Additional files

Additional file 1: Figure S1. Western blotting of rhA1AT in transiently transfected CHO-DG44 cells. CHO-DG44 cells were transiently transfected with pAID and pAIDp vectors. Both cell lysates and culture supernatants were harvested for Western blotting. Culture supernatant from a CHO-DG44 cell line that was not transfected with rhA1AT vector was used as the negative control. Additional file 2: Figure S2. Growth and rhA1AT production curves of MTX amplified rhA1AT cell pools. Adapted rhA1AT cell pools from each vector set at 50 nM and 300 nM MTX were seeded in 40 ml of serum-free medium at a cell density of 4 × 10^5^ cells/ml in 125 ml shake flask on shaker platforms set at 110 rpm in a humidified incubator at 37°C with 8% CO_2_. Cell densities and viabilities were determined daily using an automated cell counter and culture supernatant was sampled daily for analysis by ELISA to determine rhA1AT titer.

## Abbreviations

ARE: AU-rich elements
CHO: Chinese hamster ovary
CAI: Codon adaptation indexes
Dhfr: Dihydrofolate reductase
ELISA: Enzyme-linked immunosorbent assay
EMCV: Encephalomyocarditis virus
FBS: Fetal bovine serum
FRT: Flp Recombination Target
GOI: Gene of interest
hA1AT: Human alpha-1-antitrypsin
HRP: Horse radish peroxidase
HT: Hypoxanthine and thymidine
-HT: HT-deficient
IRES: Internal ribosome entry site
IVC: Cumulative integrated viable cell number
IVCD: Integral of viable cell density
μ: Specific growth rate
MARs: Matrix attachment regions
MODC: Murine ornithine decarboxylase
MTX: Methotrexate
N: Viable cell density
N_0_: Initial viable cell density
P: Cumulative rhA1AT produced
pcd: Pg per cell per day
q_p_: Specific protein productivity
qPCR: Quantitative polymerase chain reaction
rhA1AT: Recombinant human alpha-1-antitrypsin
t: Culture time
t_D_: Cell doubling time
TMB: 3,3′,5,5′-Tetramethylbenzidine
UCOE: Ubiquitous chromatin opening element
V: culture volume
