# Supplementary figures and images for: Engineering selection stringency on expression vector for the production of recombinant human alpha1-antitrypsin using Chinese Hamster ovary cells
Source: BMC Biotechnol. 2015 Jun 2;15:44. doi: 10.1186/s12896-015-0145-9 (PMC4450478; doi:10.1186/s12896-015-0145-9)

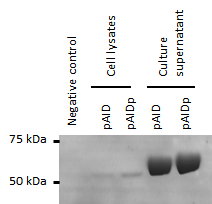

Supplement: Additional file 1: Figure S1. — Western blotting of rhA1AT in transiently transfected CHO-DG44 cells. CHO-DG44 cells were transiently transfected with pAID and pAIDp vectors. Both cell lysates and culture supernatants were harvested for Western blotting. Culture supernatant from a CHO-DG44 cell line that was not transfected with rhA1AT vector was used as the negative control. [file 12896_2015_145_MOESM1_ESM.tiff]

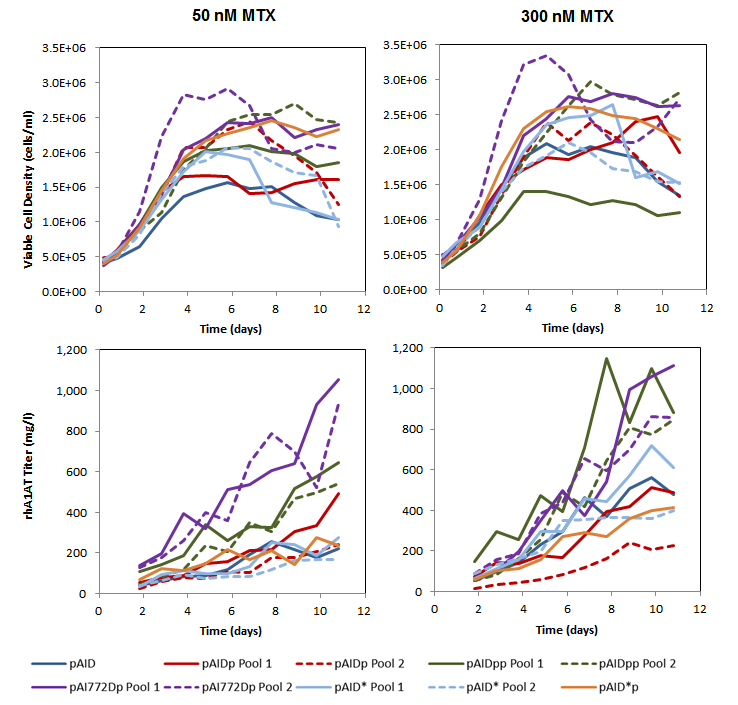

Supplement: Additional file 2: Figure S2. — Growth and rhA1AT production curves of MTX amplified rhA1AT cell pools. Adapted rhA1AT cell pools from each vector set at 50 nM and 300 nM MTX were seeded in 40 ml of serum-free medium at a cell density of 4 × 10^5^ cells/ml in 125 ml shake flask on shaker platforms set at 110 rpm in a humidified incubator at 37°C with 8% CO2. Cell densities and viabilities were determined daily using an automated cell counter and culture supernatant was sampled daily for analysis by ELISA to determine rhA1AT titer. [file 12896_2015_145_MOESM2_ESM.tiff]
